# Advanced Marjolin's ulcer of the scalp in a 13-year-old boy treated by excision and free tissue transfer: Case report and review of literature

**DOI:** 10.4103/0970-0358.53020

**Published:** 2009

**Authors:** M. Daya, T. Balakrishan

**Affiliations:** Department of Plastic and Reconstructive Surgery, Nelson R Mandela School of Medicine, University of Kwazulu-Natal, Durban, South Africa

**Keywords:** Marjolin's ulcer, Latissimus dorsi flap, Scalp defects

## Abstract

Marjolin's ulcer originates in chronic scars and wounds of diverse origin. This relatively rare tumour is most commonly a squamous cell carcinoma. The reason for malignant transformation is not well understood. The burn scar is a common predilection. We present the youngest patient in the literature, a 13-year-old male with a locally advanced squamous cell carcinoma of the scalp with intracranial extension following an unhealed burn injury at the age of three. Bilateral cervical lymphadenopathy was also noted. The tumour was excised and the large defect overlying the brain was covered by free latissimus dorsi musculocutaneous flap. At four weeks a therapeutic bilateral selective neck dissection was done. Adjuvant chemotherapy was administered. This report reiterates the importance of early diagnosis. Free tissue transfer further enhances our ability to cover complex defects associated with excision of advanced lesions.

## INTRODUCTION

Marjolin's ulcer originates in chronic scars and wounds of diverse origin. The tumour is most commonly a squamous cell carcinoma. The reason for malignant transformation is largely unknown. Usually seen in a post burn scar, the average duration of malignant transformation is 31 years. We present a case report of an advanced squamous cell on the scalp in a 13-year-old male following a burn injury with a 10-year lag interval. It is our impression that this patient may be the youngest reported case with a Marjolin's ulcer in the literature.

## CASE REPORT

A 13-year-old male presented with a fungating lesion on the scalp. He had sustained flame burns at the age of three. The lesion was never grafted and failed to heal completely. The primary care centre continued to manage the wound conservatively with frequent dressings. Eventually, a biopsy was performed and a well-differentiated squamous cell carcinoma was confirmed. He was referred to our tertiary centre for further management.

**Figure 1:**
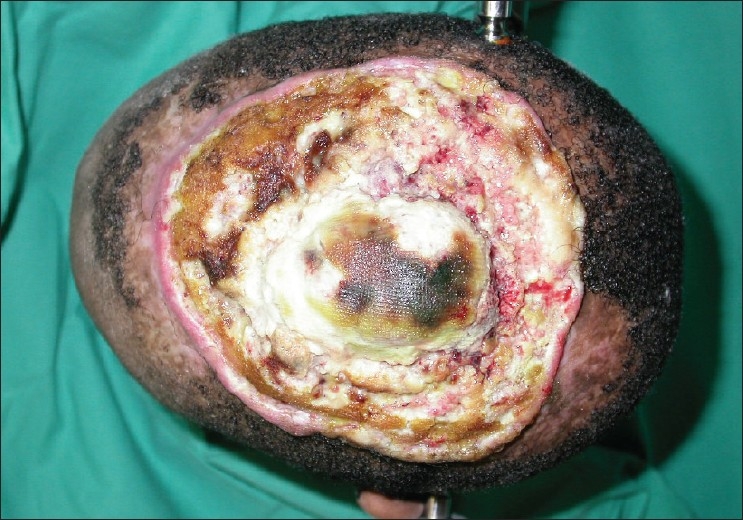
A top view of the aggressive squamous cell carcinoma of the scalp

His main complaint was pain and a foul-smelling ulcer with a mucopurulent discharge. Specifically, the patient did not give any history suggestive of epilepsy or personality change. He was noted to be underweight for his age. On examination the lesion occupied the entire central vertex of the scalp [Figure 1]. The tumour measured 12 × 15cm. The central portion of the lesion was pulsatile. Bilateral cervical lymphadenopathy was present. On neurological examination no deficits were identified. No abnormalities were noted on the cardio-respiratory and abdominal examination. Blood investigations revealed microcytic anaemia and hypoalbuminaemia. The human immunodeficiency virus serology was negative.

**Figure 2:**
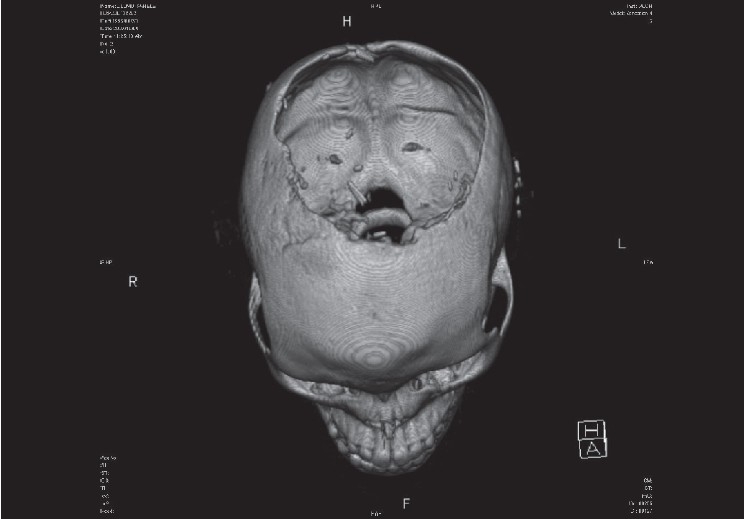
CT scan showing a large cranial bone defect due to erosion by tumour

**Figure 3:**
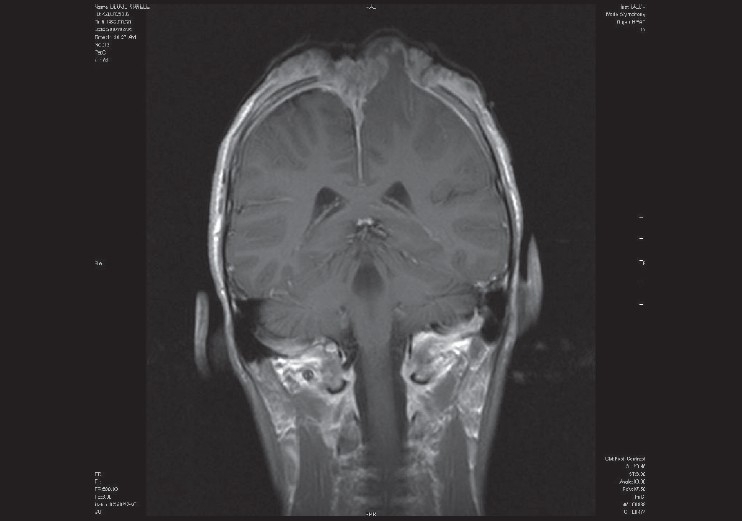
MRI scan showing tumour infiltration of the left cerebral hemisphere and the falx celebri

Investigations included a skull radiograph, computed tomography (CT) and magnetic resonance imaging (MRI). These revealed extensive tumour infiltration of the soft tissue with underlying full thickness bilateral parietal bone erosion [Figure 2]. The depth of the infiltration included the dura and the cortex of the left cerebral hemisphere with underlying oedema of the brain [Figure 3]. A filling defect in the superior sagittal sinus indicated a thrombus. A chest roentegram and an abdominal ultrasound excluded metastatic disease in the lungs and liver respectively.

The cancer was staged clinically as T4N1M0. With Neurosurgical assistance the resection of the lesion was planned.

**Figure 4:**
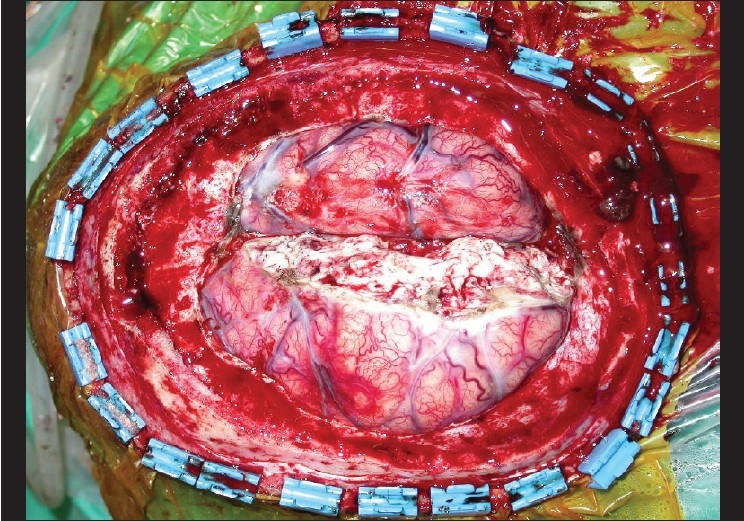
An intraoperative view following excision including the cranial bone, dura, falx celebri with the thrombosed superior saggital sinus and involved brain tissue

At surgery, the scalp, the involved cranial bones and dura were excised with 2cm tumour free peripheral margins. A suction-assisted resection of the involved cerebral cortex was done to the depth of normal brain tissue. The soft tissue defect measured 20 × 17 cm [Figure 4]. Our plan was to cover the defect with the anterolateral thigh flap. The tensor fascia lata was to serve as vascularised dural substitute. The flap harvest was abandoned after suitable calibre perforators could not be identified. A latissimus dorsi musculocutaneous flap was then used for cover. Lyoplant^®^ (bovine pericardium) was used as a dural substitute. The flap was anastomosed to the superficial temporal vessels.

Histological diagnosis confirmed an invasive acantholytic squamous cell carcinoma, an aggressive pathological type which is poorly differentiated, extending into the deep dermis and subcutaneous tissue. The final histopathology result differed from the initial biopsy result due to a larger sample size of the former as well as poorly differentiated type cells were noted within the centre of the lesion. Abundant scar tissue and perineural invasion was noted with no vascular involvement. Brain and dura confirmed the presence of tumour in the deep resection margin. The interpretation of tumour clearance was inconclusive in view of the method of resection of the involved brain tissue.

**Figure 5:**
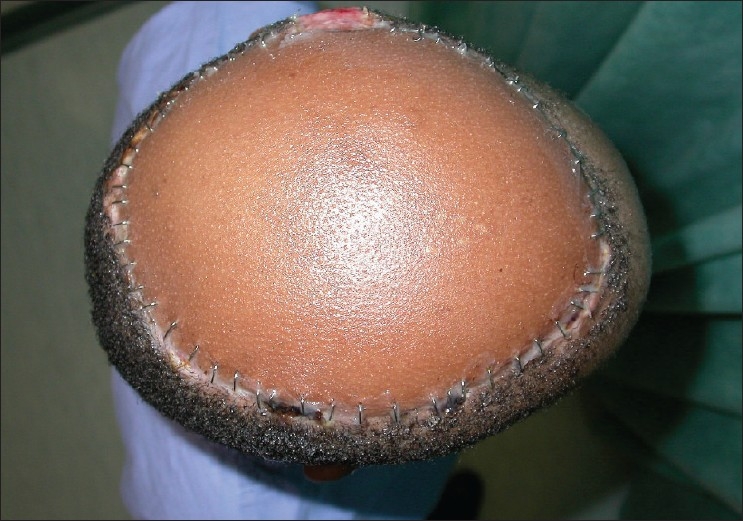
Bird's eye view at two weeks post reconstruction with a latissimus dorsi musculocutaneous flap

The patient's postoperative recovery and wound healing was uneventful [Figure 5]. The patient had a near total right-sided hemiplegia of immediate onset following the surgery. Four weeks postoperatively, the bilateral cervical lymph nodes showed no signs of regression. Instead, the distribution and the size of the nodes had increased. A fine needle aspiration biopsy confirmed the presence of metastatic disease. Therapeutic bilateral selective cervical lymph node dissection, levels 1-5, was performed five weeks after the primary excision. Histology confirmed metastases to eight out of 90 lymph nodes. The patient was referred to the oncologists for further management. Three months later a large lymph node was detected in the left pre-auricular region which was excised and confirmed as metastatic disease. Chemotherapy was advised and six cycles of Taxotere and Cisplatin were administered. At nine months post surgical excision there was no evidence of local recurrence or metastatic disease. The patient defaulted on the subsequent planning of radiotherapy to the brain. At one year he presented to the oncologist with a history of seizures and weakness in the right upper and lower limb. A scalp recurrence was also noted. A CT scan of the brain showed left intraparenchymal parietal lobe mass lesion with solid and cystic components. The lesion was not amenable to neurosurgical intervention. The patient was offered palliative radiotherapy to the whole brain at a dosage of 3Gy each over nine cycles. The response was poor and the patient was discharged to the care of a hospice resulting in his demise three months later.

## DISCUSSION

Marjolin's ulcer is a rare and often aggressive cutaneous malignancy that arises in previously traumatized, degenerated and chronically inflamed skin or scar tissue particularly after burns.[1] Other wounds of diverse origin include chronic fistulae, chronic radiation dermatitis, chronic osteomyelitis, chronic venous ulcers, discoid lupus, pilonidal sinus and even operative scars.[2,3] In 1828, Jean Marjolin first described a tumour arising in burn scars but did not recognize its malignant nature.[2] However, his name is now synonymous with a variety of cancers arising in any scar tissue or chronic wounds.

The commonest histological tumour type is a squamous cell carcinoma.[1,2] Less common in order are basal cell carcinomas, melanomas and sarcomas of fibrous tissue, fat or bone origin.[1,2,4] The exact incidence of malignant transformation is unknown[2,4,5] and is perhaps dependent on the cohort group that is being studied.[6] Various studies indicate that Marjolin's ulcers make up 1.2% of all skin cancers, 2% of squamous cell carcinomas and 0.5% of basal cell carcinoma.[2,4] It is thought that basal cell carcinomas occur when the burn is more superficial and the hair follicles and sebaceous glands are spared. Radiation burn dermatitis is the only injury that has a higher predilection to basal cell type carcinomas.[2,4]

The reported patient is an African male. Dark skinned individuals are not prone to solar skin cancers,[2] but a traumatic burn with healing by secondary intention is noted to be a significant factor in our case report.[2,5,7] Most burn scar carcinomas occur in patients who were not grafted or in areas of graft failure.[2,8] There is less frequent malignant transformation in burns that have been excised and grafted.[4] Greatly hypopigmented and thickened scars are more likely to progress into a malignant lesion.[4] Heat injury and sun exposure also predispose to malignant transformation. The harsh subtropical climate of South Africa may be a significant factor in the pathogenesis of this lesion.[4,8]

Marjolin's ulcers occur at any age and in all races.[2,4,5] Men are more commonly affected than women with a ratio of about 3:1. The average age of onset is 58 years.[4] In the literature 18 years of age is reported as the youngest case and 84 years[9] the oldest. The lag time from injury to onset of the cancer varies from four weeks[10] to 68 years.[6] The median time lag being about 31 years.[2] Lag time is inversely proportional to the age of the patient at the time of the injury;[2,4,6,10] however, this trend does not always exist.[10] Our patient demonstrated a time lag of 10 years or less because the actual time of onset could not be ascertained. The advanced stage at which this patient presented may be suggestive of a significantly earlier onset.

The reasons for malignant transformation are largely unknown.[4,6] Virchow suggested that every cutaneous scar subjected to continuous irritation has the increased potential for malignant transformation.[2,4] It is generally accepted that neither the burn nor chronic scar is in itself carcinogenic.[9] Some of the suggested mechanisms are:

1. Sequestration of the chronic ulcer by the scar tissue such that immunosurveillance by the lymphatic system is compromised and tumour-specific antigens escape detection.[4–6,10,11]
2. Low T-cell counts.[5]
3. Genetic factors like mutations of the FAS-APO-1/CD95 genes in burn scars which is not detected in other squamous cell carcinomas.[2,9]
4. Decreased vascularity of the scar.[4–6]
5. Injury-related release of toxins and activation of preneoplastic cells by co-carcinogens.[2,6,10]
6. Infection may serve as a co-carcinogen.[2,4,12]
7. Decreased regenerative capacity of a burn scar.[4,6]

Some of these mechanisms may be appropriate in our reported patient. He came from an impoverished socioeconomic background and therefore malnutrition may be a contributing factor. Specifically, human immunodeficiency virus that compromises the T-cell function was excluded.

Risk factors for malignant change can be summarized to include healing by secondary intention, non-healing secondary to sepsis, fragile and easily traumatized tissue, ulcerated scars with obliterated lymphatics, and poor local and systemic immune resistance.[9] Managing these risk factors is the key in prevention of Marjolin's ulcers.

The prevalence of this lesion is low and therefore there has been general disagreement regarding its clinical features, methods of treatment and prognosis.[2,4]

Identification of risk factors and a high index of suspicion is the key to early diagnosis.[4,7,9,12] In the clinical presentation, noting a change in the sensation or appearance of the scar or ulcer mandates a histological assessment. As with most tumours, early treatment renders the best prognosis.[7,13] In our patient, the health system can be heavily criticized for failing to recognize the disease process in a patient who presented regularly for treatment.

Treatment modalities include wide local excision,[14] block dissection of the regional nodes, amputation in advanced lesions of limbs,[2,4,9,12,14] radiotherapy and chemotherapy.[2,4,9]

The mainstay of treatment is wide local excision with a margin of 2 cm.[1–4,12] It is imperative that the surrounding scar tissue be excised. Frozen sections are useful when vital structures border the resection margins.[2,7] At times, irregular scar edges and surfaces need to be excised with larger margins using appropriate surgical judgment.[2,13] If at all possible, clear margins are the best attempt at cure.[2] Recurring ulcers should be excised even if they are not malignant.[7]

The choice of soft tissue cover for the defect follows the principles of oncoplastic surgery.[7] The choice for a free tissue transfer in this patient was dictated by the size of the defect,[15] age of patient, potential need for radiotherapy[7] and availability of microsurgical expertise. Our first choice, the anterolateral thigh flap for the reconstruction was apt because the fascia lata was a suitable substitute for the dura and the donor site morbidity was acceptable. The latissimus dorsi musculocutaneous is traditionally one of the best options for reconstruction of large scalp defects. However, perforator flaps, size permitting, do provide for adequate cover without the need for the sacrifice of a major muscle.

Prophylactic regional lymph node dissection is controversial.[2,4,7,10,16] Lymph nodes may be enlarged because of chronic infection or metastases.[6] The size of lymph nodes is not a dependable predictor of malignancy. Elective lymph node dissection may be indicated in high-risk tumours in lower extremities[8,14] where metastases are reported to approach 54%.[10] Some authors recommend lymph node dissection only in the presence of clinically positive nodes.[5,8] Fitzgerald suggested that lymph node excision should be done only if there is no decrease in the size of the lymph node three months after excision of the malignant ulcer. Bostwick recommends local excision followed by elective regional lymph node dissection two to four weeks later.[5,10] No consensus has been reached.[2,4–6] Our main reason for ignoring the lymph nodes in the initial surgery was due to the complex primary resection of the tumour and reconstruction. There was also a very high risk of the nodes being reactive due to the long-term suppuration associated with the ulcerated tumour. It was only after a successful surgical outcome and the increase in the growth and number that we decided to turn our attention to management of the cervical nodes. We are not clear of the impact of the block dissection on the survival rate in this patient and the relevance of 8 out of 90 neck nodes on the prognosis.

Little prospective data is available on the role of radiotherapy. Primary radiotherapy has not been effective.[2,4,12] Its role is therefore palliation. Adjuvant radiotherapy should be given for incomplete tumour margins[4,6,9,12] and possibly for the lymphatic basin following a positive node dissection.[2]

Chemotherapy - 5 Fluorouracil and Cisplatin have been advocated both topically and intralesionally for primary treatment and decreasing tumour size. Some centres reserve it for patients with a poor prognosis or distant metastases.[11]

Recurrence rates are high despite current treatment advances and appropriate long-term follow-up is imperative. Recurrences are almost always local but metastases to the lung, brain, liver and distant lymph nodes have been reported.[6,14] Most series indicate that the incidence of recurrence is in the range of 20-50%.[2,4] In our patient the excision was surgically and histologically incomplete and therefore required adjuvant radiotherapy. A default of 3 months of radiotherapy exascerbated the failure of local control at 12 months.

Prognosis of Marjolin's ulcer is poorly documented. It is related to the local extent of disease, location, pathological types and degree of differentiation, depressed immune systems, latency period and most importantly lymph node metastases. The squamous cell carcinoma of Marjolin's origin is locally and systemically more aggressive than other forms of squamous cell carcinoma.[1,2,4,8,12] Like the pathogenesis and aetiology of Marjolin's ulcers, the mechanisms of lymphatic spread are both contentious and unexplained. Scar tissue does act a barrier for lymphatics[10,11] and only once this barrier is compromised either by tumour excision or local spread then invasion of normal lymphatic tissue occurs. The lack of lymphatics in the scar tissue provide a sanctuary for the tumour cells in them from the host's immune system. Scanty blood supply because of fibrosis renders pharmacological sanctuary to the cancer cells from systemic chemotherapeautic agents. The incidence of metastasis ranges from 10% for a low-grade tumour to 86% for a poorly differentiated tumour.[2] Patients with nodal metastasis have a five-year survival of 35%.[2] Once visceral metastases are present the five-year survival rate was reported to be 25.1%. Marjolin's ulcers of the lower limb have the worst prognosis. The face and neck have the best five-year survival rate of 50-70%.[2] Survival rate is the best for patients who are disease-free without metastases for three years.[16] A local recurrence after complete excision signifies the aggressive nature of the tumour and further control remains challenging. Basal cell carcinomas have a relatively better prognosis than squamous cell carcinomas.[10]

## CONCLUSION

A delay in diagnosis remains a major stumbling block in improving the outcome for patients with Marjolin's ulcers. The patient's complacency towards a longstanding chronic wound and the doctor's low index of suspicion are major contributing factors. Age is not an exclusion criterion. Malnutrition and UV exposure contribute significantly to the pathogenesis of Marjolin's ulcers. A delayed diagnosis results in the need for more extensive surgery and an increased risk of metastatic spread.[6,8] The availability of microsurgical expertise makes wide local excision of advanced lesions possible,[8] be it curative or palliative. The latissimus dorsi flap is a traditional workhorse for major scalp reconstruction which can reliably cover the entire scalp. Surgery is the primary treatment for local control and radiotherapy has an adjunctive role that has to be backed by careful oncological surveillance. However, in this patient despite the failure in local control and his eventual demise, the surgical management rendered a better quality of life in the interim period.
